# A Printed Reconfigurable Monopole Antenna Based on a Novel Metamaterial Structures for 5G Applications

**DOI:** 10.3390/mi14010131

**Published:** 2023-01-03

**Authors:** Saba T. Al-Hadeethi, Taha A. Elwi, Abdullahi A. Ibrahim

**Affiliations:** 1 Electrical and Computer Engineering Department, Graduate School of Science, Altinbas University, Istanbul 34217, Turkey; 2 International Applied and Theoretical Research Center (IATRC), Baghdad 10001, Iraq

**Keywords:** MTM, TR, monopole, 5G, BER

## Abstract

A novel antenna structure is constructed from cascading multi-stage metamaterial (MTM) unit cells-based printed monopole antenna for 5G mobile communication networks. The proposed antenna is constructed from a printed conductive trace that fetches four MTM unit cells through four T-Resonators (TR) structures. Such a combination is introduced to enhance the antenna gain-bandwidth products at sub-6GHz bands after exiting the antenna with a coplanar waveguide (CPW) feed. The antenna circuitry is fabricated by etching a copper layer that is mounted on Taconic RF-43 substrate. Therefore, the proposed antenna occupies an effective area of 51 × 24 mm^2^. The proposed antenna provides an acceptable matching impedance with S_11_ ≤ −10 dB at 3.7 GHz, 4.6 GHz, 5.2 GHz, and 5.9 GHz. The antenna radiation patterns are evaluated at the frequency bands of interest with a gain average of 9.1–11.6 dBi. Later, to control the antenna performance, four optical switches based on LDR resistors are applied to control the antenna gain at 5.85 GHz, which is found to vary from 2 dBi to 11.6 dBi after varying the value of the LDR resistance from 700 Ω to 0 Ω, in descending manner. It is found that the proposed antenna provides an acceptable bit error rate (BER) with varying the antenna gain in a very acceptable manner in comparison to the ideal performance. Finally, the proposed antenna is fabricated to be tested experimentally in in free space and in close to the human body for portable applications.

## 1. Introduction

Recently, mobile communication networks were developed rapidly to satisfy the current mobile communication services [1]. However, the evolution of current mobile communication systems requires high data rates in the range of Giga bits per second [2]. Thus, the need to develop modern antenna designs to serve the current wireless communication systems became one of the most urgent essentials [3]. In such cases, enormous challenges face scientific researchers to develop antennas with a wideband and enhanced gain due to the antenna size limitations [4]. However, many researchers succeeded to overcome the motioned challenges by invoking several techniques. For example, the authors in [5] designed a composite right-left-hand microstrip antenna with an enhanced gain-bandwidth product for RF energy harvesting [6] at 2.45 GHz and 5.8 GHz. A low-profile fractal antenna structure based on MTM array was developed for modern Wi-Fi systems [7]. In [8], a circularly polarized fractal patch of Koch-snowflake 1st iteration with ground plane MTM defects was proposed for 5G handsets at 3.5 GHz only [9].

In another aspect, MTM played a major role in developing the antenna systems due to the unusual properties based on exceptional electromagnetic constitutive parameters [10]. Therefore, the authors in [11] used certain MTM inclusions on their antenna patch to control the beam-steering capacity by using two semiconductor switches. Another design was proposed in [12] based on using an MTM layer on the top of an antenna patch to enhance the antenna gain at Wi-Fi bands. The introduction of the MTM structure to the antenna ground plane was developed in [13] to minimize the back lobe effects at 3.4 GHz. Another MTM introduction was proposed in [14] to ensure the antenna surface wave propagation toward the end-fire direction for gain-bandwidth enhancements. Finally, a design of MTM antenna was proposed in [15] to realize a gain enhancement at 4.6 GHz only for 5G communication networks.

In [16], a microstrip antenna array was presented for 5G networks at 28 GHz bands-based 4 × 4 elements; however, the selected band was suffering from a limited propagation that was not considered in their work. Another design was proposed in [17] based on using a triple-band with dual polarization printed circuit antenna array for MIMO systems in the sub-6GHz for 5G applications. An antenna array was developed for 5G systems by orienting four antenna elements on a 3D profile and separated with MTM defects; such an antenna provided a high gain-bandwidth product. However, the proposed antenna in [18] was very complicated and not applicable with low-profile devices [19]. Moreover, another design was proposed in [20] based on a reconfigurable antenna element with optical switches but without considering the array systems analysis. In [21], a design of a microstrip antenna based on MTM defects was proposed for the same application with a limited gain at a single frequency band.

In this work, an antenna design based on a novel MTM array is proposed to realize a miniaturized antenna structure for 5G communication networks. The proposed MTM arrays are mounted in parallel to a conductive trace to ensure in-phase reflection characteristics and high-impedance response against the effects of the surface wave retardation from the antenna edges. Such technique could realize gain-bandwidth enhancements over the conventional printed antenna structures as will be proven later. In Section 2, the antenna geometrical details are presented. The antenna design methodology is discussed in Section 3. In Section 4, the obtained results are validated. Finally, the paper is concluded in Section 5.

## 2. Antenna Design and Geometrical Details

**Figure 1:**
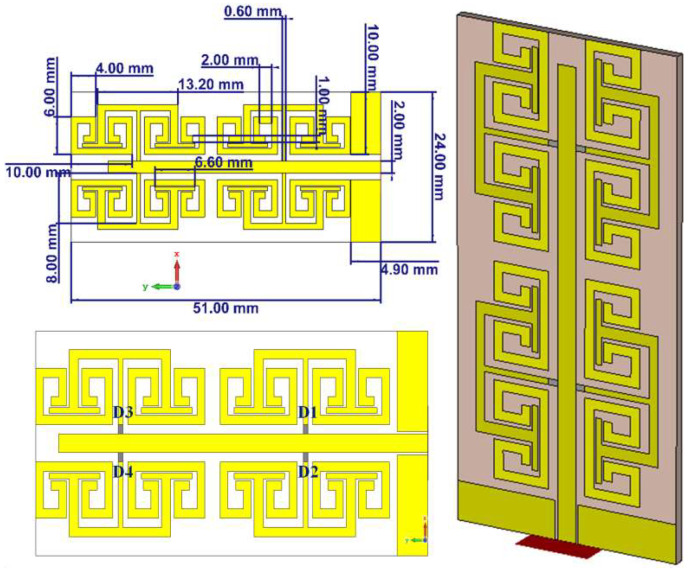
The proposed monopole antenna geometrical details.

The proposed antenna is structured from CPW to feed a microstrip line as seen in Figure 1. The microstrip line is invoked to excite TR, which is coupled to a single split ring resonator (SSR) unit cell [7]. The proposed SSR is invoked to reduce the antenna length by exciting the harmonic plasmonic surface waves that increase the effective electrical antenna length [4]. The proposed TR structures inductively excites the proposed SSR to increase the antenna bandwidth significantly in comparison to those based on direct connection [9]. Each unit cell is constructed based on a fractal series of size reduction that increases the current path motion on the patch surface [10]. In the same aspect, the antenna size is miniaturized to λ/5. The transmission line is designed with a width of 2 mm to realize 50 Ω match circuit and to transfer the surface current motion to the proposed TR [7]. The coupling effects on the proposed TR are applied to force the current movement toward the proposed SRR as explained in [6]. The proposed CPW technique is introduced to avoid surface wave retardation that creates opposite interferences and inductive losses due to the use of the transmission line junction. These effects can be removed by the capacitive effects of the matching circuit [8]. The proposed TR is introduced to suppress the surface waves from the antenna edges, which has a negative effect on the radiation efficiency [9]. The microstrip patch is printed on Taconic RF-43 substrate without a ground plane and dielectric constant of 4.3 with a height of 1.67 mm.

## 3. Design Methodology

In this section, the proposed antenna dimensions are studied parametrically to realize the effects of varying them with respect to the antenna performance. The parametric study is conducted using a numerical analysis based on CST MWS [3]. The proposed antenna is structured from four unit cells with CPW feed as seen in Figure 1. The proposal patch is printed on the substrate without a ground plane. The cell geometry is considered to increase the effective length within a limited area. The proposed TR has the advantage to increase the bandwidth and support the antenna to reduce the patch losses [15]. The transmission line is used to transfer the power from the RF source to the antenna part. The matching load is used to match the antenna radiation to the free space impedance [7]. The proposed CPW feed is considered to match the power over a wide range of frequencies with low dispersion effects and phase retardation [9].

### 3.1. Microstrip Line Design

**Figure 2:**
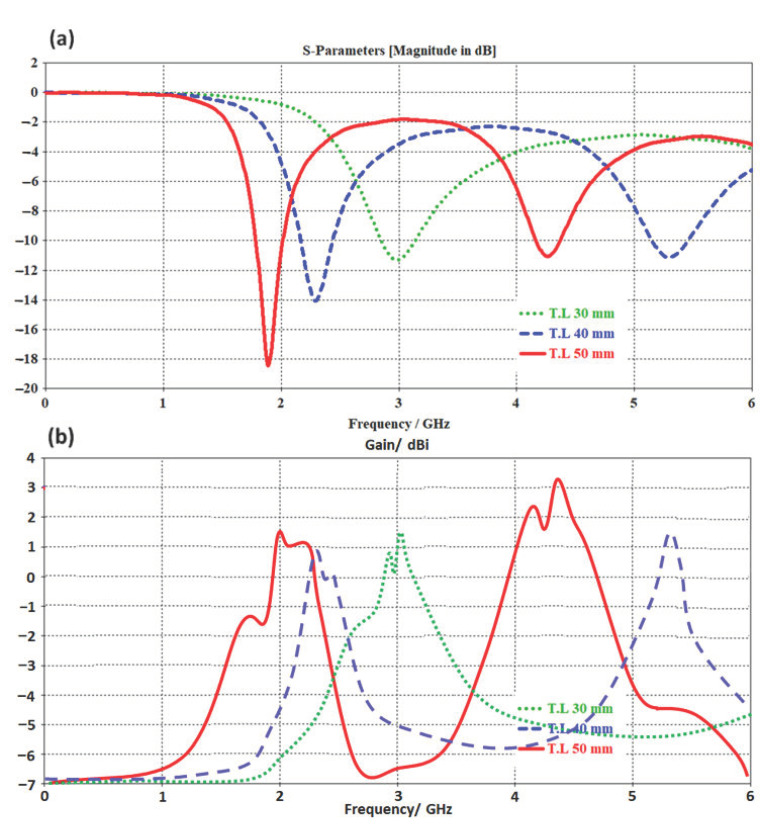
Antenna performance with changing L value: (**a**) S_11_ and (**b**) gain spectra.

For parametric analysis purposes, the authors changed the transmission line length from 30 mm to 50 mm with respect to monitoring S_11_ and gain spectra. Such analysis is conducted to ensure the effects of changing the transmission line length on the antenna performance. Figure 2a shows the evaluated S_11_ spectra for three considered microstrip lengths. It is found that by increasing the transmission line length, the antenna frequency resonance shifts toward the lower frequency bands due to the increase in the electrical length [10]. Nevertheless, the antenna gain is found to increase rapidly with increasing the antenna length due to the fact of increasing the surface current [11]. Therefore, the authors considered the transmission line with a length of 50 mm as the best choice because it showed two frequency resonances at 1.9 GHz and 4.3 GHz with S_11_ of −18.4 dB and −11 dB and gain of 1.5 dBi and 3.1 dBi as in Figure 2b.

### 3.2. Unit Cell Effects

**Figure 3:**
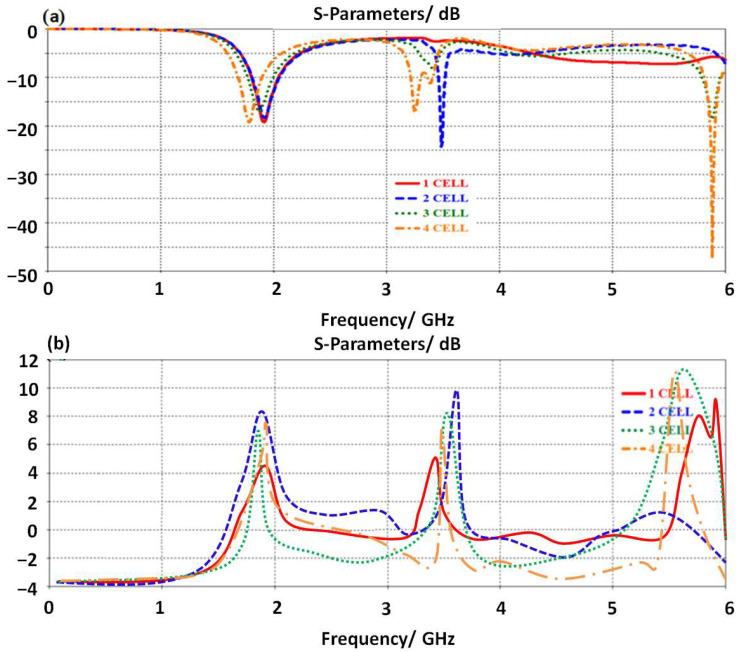
Antenna performance with different cell numbers: (**a**) S_11_ spectra and (**b**) gain.

The proposed antenna design is structured from SRR geometry with four unit cells to form a butterfly shape. Therefore, to study the effects of adding them to the antenna performance, the authors applied a parametric study-based CST MWS. In this simulation, the authors included the first unit cell in the antenna design, then compared it to the obtained results, in terms of S_11_ and gain spectra, from the identical antenna design based on (two, three, and four) unit cells. We found, at the first cell, the antenna shows multiple frequency bands within the frequency range of interest as shown in Figure 3a. By increasing the number of the unit cell, a clear increase in the antenna resonance modes is observed. This is due to the fact of fading the current motion at the junction points between the transmission line and the unit cells. This fading is due to the current chocking by the inductive addition from the proposed unit cells [6]. Therefore, the antenna gain is found to be increased rapidly, due to the parallel connection between the fading points that creates an excellent electromagnetic aperture coupling with the free space impedance [12]. This is achieved due to the fact of the connection between the unit cells on the proposed transmission line serially. In such a connection, the current motion is mostly in the same direction as the proposed unit cells. Also, adding the unit cell in this manner, two parts opposite to each other from the transmission line, creates antiparallel equalized forces that add nothing to the current motion [12] to keep the first frequency resonance at 3.5 GHz. Nevertheless, the combination between them is in a series of connection results adding the effects of them directly to each other. Since the proposed unit cell is capacitive inclusion, adding them and increasing their number may realize a significant reduction in the equivalent capacitor [8], which in turn increases the particular operating frequency band [9]. Therefore, the antenna gain is found to be about 5.5 dBi at 3.5 GHz as seen in Figure 3b.

### 3.3. TR Effects

**Figure 4:**
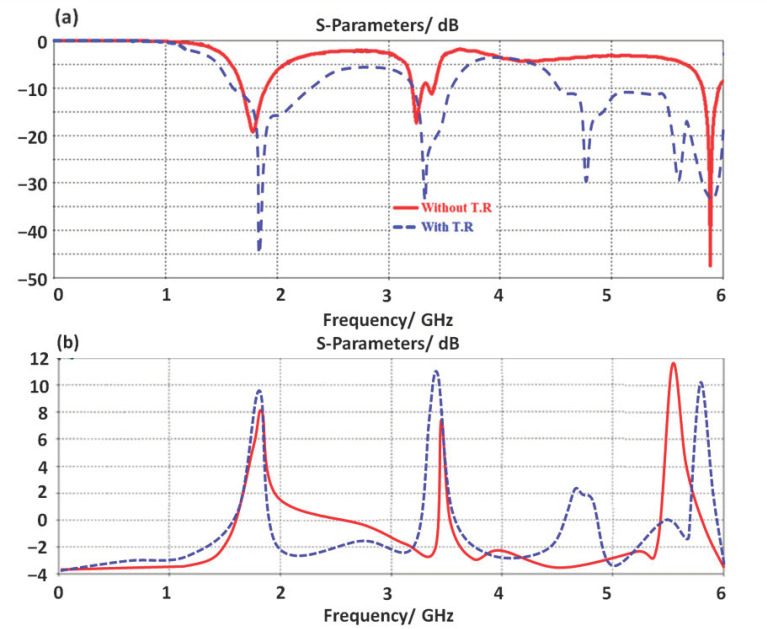
Antenna performance with and without TR structure: (**a**) S_11_ spectra and (**b**) gain.

This section shows that the effects of the proposed TR introduction on the antenna performance are characterized numerically. It is conducted to couple the electromagnetic energy inductively at the resonance frequency [5]. It is found that the proposed TR realizes a bandwidth enhancement due to the effects of the inductive coupling that stores the accumulated charges on the edges of the conductors [11]. Therefore, the antenna bandwidth is enhanced with an insignificant reduction in the antenna gain as shown in Figure 4a. The antenna gain at this stage is found to be increased up to 6.8dBi after introducing the proposed TR structure. Such an increase in the antenna gain is attributed to the charge accumulation cancelation by the inductive part of the proposed TR structure [7].

### 3.4. CPW Effects

**Figure 5:**
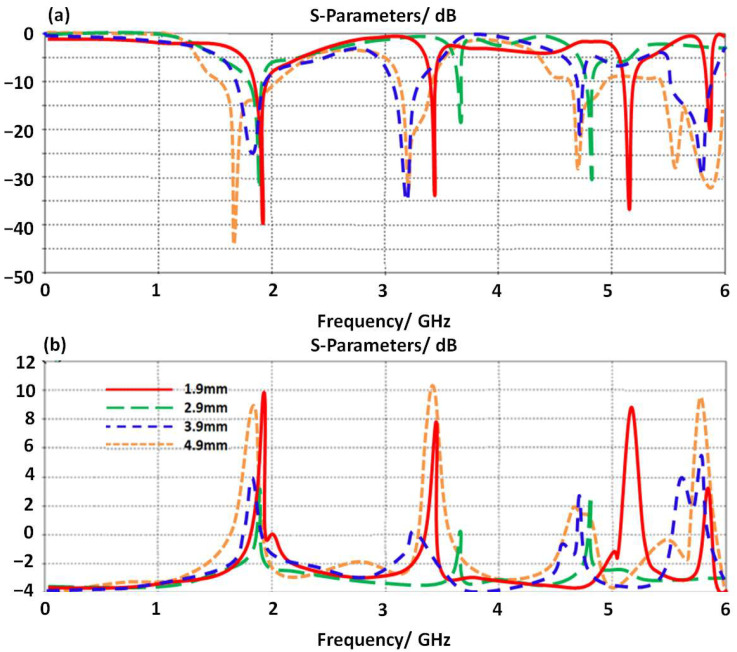
Antenna performance varying the ground plane length: (**a**) S_11_ spectra and (**b**) gain.

The proposed antenna is fed with a CPW structure as shown in Figure 1. Therefore, to optimize the antenna performance, the authors applied a study to realize the effects of varying the proposed CPW dimensions on the antenna performance in terms of S_11_ and gain spectra. As shown in Figure 5a, the antenna shows a significant enhancement by changing the distance between the antenna patch and the ground plane part of the proposed CPW. The antenna gain is found to be enhanced by reducing the air gap between the proposed patch and the proposed CPW ground plane. This is due to eliminating the effects of the electrostatic capacitive coupling between the antenna parts and the ground plane [5]. The antenna is found to provide five main bands at 1.6 GHz, 2.6 GHz, 3.9 GHz, 4.5 GHz, and 5.85 GHz with S_11_ below −10 dB at 4.9 mm distance. Nevertheless, the antenna gain is affected by various changes at different frequency bands as shown in Figure 5b. However, the antenna bandwidth at 4.9 mm distance is found to be significantly enhanced over other distances.

### 3.5. Switching Effects

**Figure 6:**
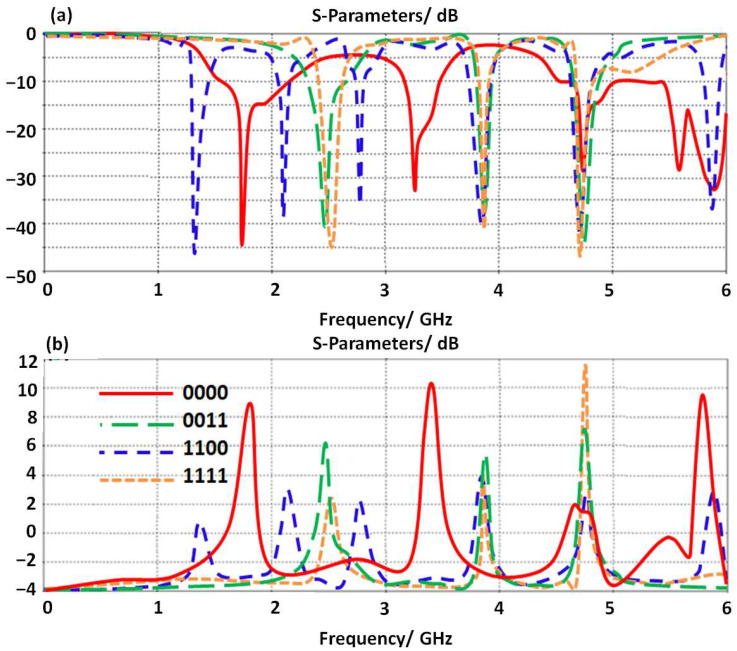
Antenna performance with different switching sceneries: (**a**) S_11_ spectra and (**b**) gain.

The proposed antenna performance is reconfigured by introducing four LDR diodes to control the frequency resonances. We found such a technique is very applicable to controlling the generated frequency bands by controlling the current motion [2]. In our study, we selected four cases only as summarized in Table 1. The antenna performances in terms of S_11_ and gain spectra are presented in Figure 6. The antenna gain is found to be insignificantly affected by changing the diode’s operation due to the fact of coupling effects between the antenna transmission line and the proposed SRR unit cells. The rest of the other cases are not considered in this study due to space limitations. It is good to mention the activation of the LDR diode by applying a biasing voltage to obtain a logic_1. In case of deactivating the LDR diode, a logic_0 can be obtained.

Another study is applied in this section; the proposed antenna performance based on LDR switches is studied by varying the LDR resistance value from 0 Ω to 700 Ω. In such a study, the proposed antenna is switched by changing the resistance level for all LDR resistors at the same time. This is because the antenna gain is found to be varying for such a study. It is found that the proposed antenna gain decays gradually, for example at 4.5, at which the gain is maximum, from 11.6 dBi to 2 dBi with increasing the resistance value as listed in Table 2. Such gain enhancement is achieved by cascading multi stages of MTM unit cells to realize a high gain at the resonant frequency. This observation is quite similar to the plasmonic resonance of the inherent resonant of the scatter structure [5]. This is due to the fact of affecting the resistance variation on the surface current motion [5]. Consequently, the values of BER are calculated according to the antenna gain at each LDR value. In such a process, the evaluated BER from R2022a MATLAB version 9.12 platform is performed by a computer simulation and numerical analysis QAM modulation scheme. The evaluated BER using a function returns a number in Matlab. The number of bits differ in the comparison and the ratio of number to the total number of bits. The function determines the order in which it compares transmitted bits to received bits based on their sizes.

The simulation is conducted by considering AGWN channel for (8, 16, 32, and 64)-QAMs. The considered AGWN is channel is generated from a random source in a MATLAB function. The maximum bit error is placed at 100 and the maximum number of bits is taken as 1 × 10^7^. The objective is to evaluate each QAM index with respect to BER against LDR resistance variation as listed in Table 2. It can be observed from the achieved results that BER decreases with the LDR value increase to perform better modulation in terms of BER with QAM order decrease.

## 4. Results Validations

**Figure 7:**
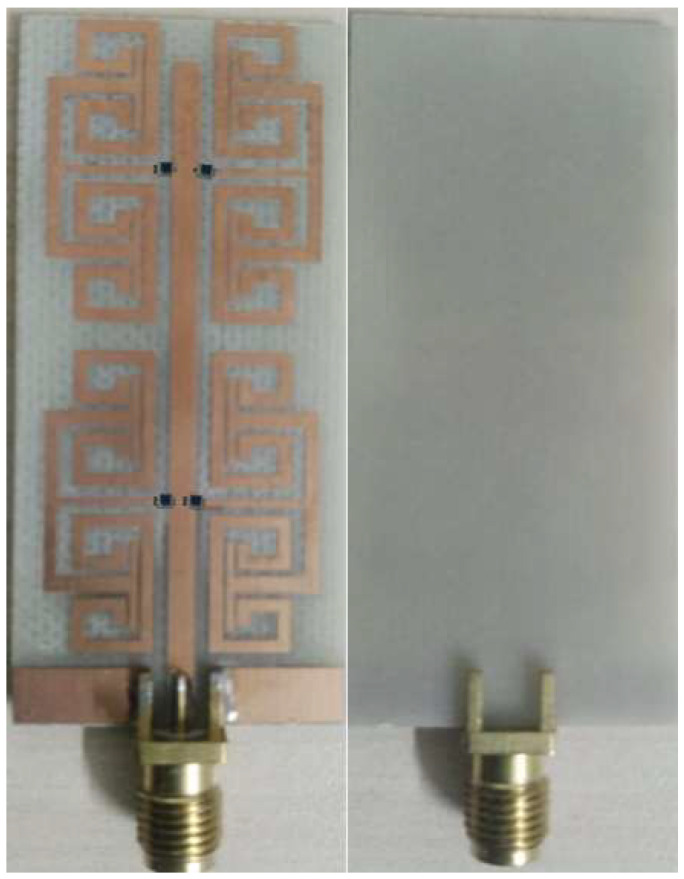
Fabricated prototype structure.

In this section, the proposed antenna results that were evaluated numerically using CST MWS are compared to measurements. The proposed antenna performances in terms of S_11_ and gain spectra are evaluated up to 6 GHz. Therefore, the proposed antenna is fabricated and tested experimentally. The antenna is fabricated using a wet chemical etching process, where the fabricated prototype is shown in Figure 7.

To give the complete meaning of portable applications of such antenna, the antenna performances are tested in two different scenarios.

### 4.1. In Free Space Environments

**Figure 8:**
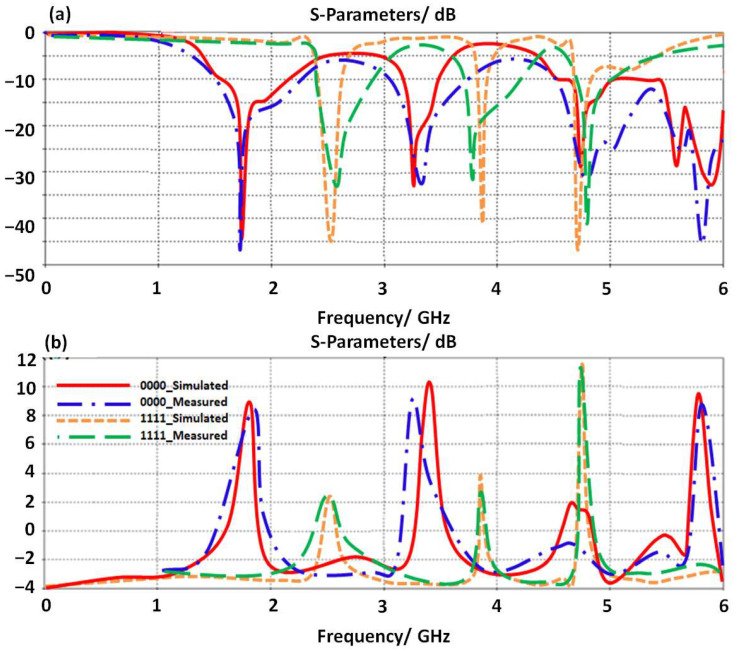
Antenna performance measurements with different switching sceneries: (**a**) S_11_ spectra and (**b**) gain.

In this section, the proposed antenna is tested experimentally to evaluate S_11_ and gain spectra at two LDR values. As shown in Figure 8a, the proposed antenna S_11_ spectra are found to show an excellent response to the switching process; when all LDR resistance values are changed from OFF status, logic_0, to ON status as logic_1. Such a process is achieved by covering all LDR resistances with lids for OFF status. For ON status, LDR resistances are lighted all without cover lids to be activated ON. Consequently, it is found that the proposed antenna gain spectrum realizes a gain of 11.6dBi when all LDR resisters are switched ON with light; however, when all LDR resistors are switched OFF, the gain decreases to 2 dBi at 4.5 GHz as seen in Figure 8b. It is found that the measurements agree well with simulated results with insignificant errors.

### 4.2. In Close to Human Body

**Figure 9:**
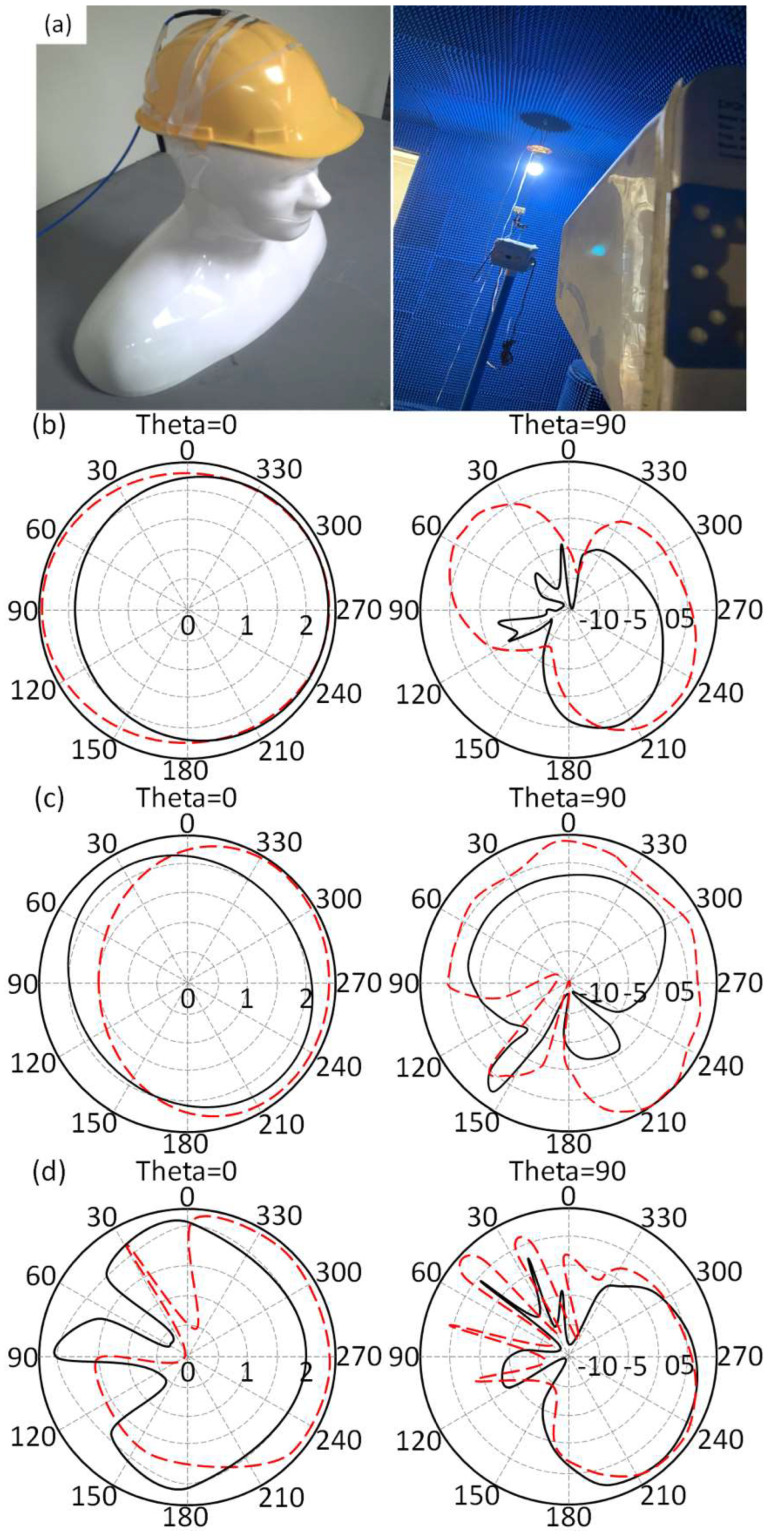
Antenna radiation pattern measurements: (**a**) Experimental setup, (**b**) 2.3 GHz, (**c**) 3.7 GHz, and (**d**) 4.4 GHz.

Later, the antenna performance in terms of S_11_ and radiation pattern is measured close to the human body and presented in Figure 9a. For this, the antenna is located directly on the human skin to measure the antenna S_11_ and gain spectra. Later, the measured results for the mounted prototype close to the human body are compared to the case of having the antenna in the free space environment. Therefore, the human phantom model is invoked to locate the antenna structure during the measurement process as shown in Figure 9b. It is found that the proposed antenna S_11_ spectrum is affected moderately after locating the antenna close to the human body as seen in Figure 9c. The measured antenna gain spectrum is shown in Figure 9d. It is found that the proposed antenna shows degradation in the antenna gain due to the effects of losses by the human tissues [4]. The antenna gain and bandwidth degradations are recorded in Table 3. In general, the degradations are found to be acceptable in the scenario of having the antenna at such a close distance to the human body.

The measured antenna radiation patterns are found to be significantly affected for the case of the antenna in a free space environment in comparison with those obtained from having the antenna close to the human body as shown in Figure 9a. It is good to mention that the antenna radiation patterns are measured with OFF statues case at 2.3 GHz, 3.7 GHz, and 4.4 GHz for both antenna location scenarios at azimuth (theta = 0°) and zenith (theta = 90°) planes.

The antenna performances in terms of frequency of operation, gain, size, and reconfiguration process is compared in detail in Table 4. It is found that the proposed antenna realizes excellent size reduction with an excellent performance in comparison to those published in the literature.

## 5. Conclusions

In this paper, the proposed antenna is designed for 5G applications at sub-6GHz bands. The antenna is structured using MTM technology to miniaturize the size up to λ/8 at 3.5 GHz. The antenna shows four operating bands with S_11_ ≤ −10 dB at 3.7 GHz, 4.6 GHz, 5.2 GHz, and 5.9 GHz with an average gain from 6.1 dBi to 8 dBi. To ensure antenna operation with a high reconfiguration mechanism, four LDR diodes are conjugated to the TR structure to control the surface current flow for frequency control. Therefore, the antenna operation and performance nominated it as an excellent candidate for 5G networks at sub-6GHz frequency bands. The antenna performance in terms of S_11_ and gain spectra along with the radiation patterns that are evaluated from the numerical simulations agrees very well with measured results. Finally, the investigated results reveal that the proposed antenna is an excellent candidate for short and medium wireless communications of portable wireless systems.

## Figures and Tables

**Table 1:** Comparison between the switching scenarios operation in terms of antenna performance.

R_1_	R_2_	R_3_	R_4_	Frequency/GHz @ S_11_ ≤ −10 dB	Gain/dBi
0	0	0	0	1.8, 3.3, 4.8, 5.5, 5.8	9.8, 10.8, 2, 0, 9.9
1	1	0	0	2.5, 3.9, 4.8	2.7, 4.1, 3.8
0	0	1	1	1.3, 2.1, 2.8, 3.9, 4.8, 5.8	1.7, 2.7, 2.3, 4, 3.5, 3.7
1	1	1	1	2.5, 3.9, 4.8	2.9, 4, 11.6

**Table 2:** BER evaluation at different LDR resistance values.

LDR/Ω	Gain/dBi	BER@QAM-8	BER@QAM-16	BER@QAM-32	BER@QAM-64
0	11.6	0.0894	0.0921	0.1164	0.1313
100	10.1	0.1034	0.1171	0.1234	0.1431
200	8.6	0.1091	0.1198	0.1391	0.1595
300	6.2	0.1178	0.1281	0.1498	0.1618
400	5.5	0.1412	0.1521	0.1609	0.1732
500	4.1	0.1569	0.1692	0.1760	0.1860
600	3.2	0.1678	0.1773	0.1802	0.1971
700	2	0.1756	0.1851	0.1977	0.2056

**Table 3:** Comparison between antenna results at two different antenna environments.

In the Free Space		On the Human Body	
Frequency/GHz	Gain/dBi	Frequency/GHz	Gain/dBi
2.5	2.9	2.3	2.1
3.9	4	3.7	3.3
4.8	11.6	4.4	9.6

**Table 4:** Comparison between this work and previous research.

Previous Work	Frequency/GHz	Antenna Ddimensions/mm^2^	Gain
[13]	5.5	21 × 17.5	3
[14]	5.01–6.12	25 × 20	3
[17]	2.5/3.5/5.5	32 × 20	1.67
[18]	2.4/5.2/5.8	20 × 22	3
[19]	3.5/5.5	35 × 39	2.2
This work	2.5, 3.9, 4.8	51 × 24	2.9, 4, 11.6
